# An interview with Lincoln Issamu Nojima

**DOI:** 10.1590/2177-6709.24.3.022-032.int

**Published:** 2019

**Authors:** Lincoln I. Nojima, Daniel Brunetto, José Valladares, Luciane Macedo de Menezes, Marco Antonio Schroeder, Mirian Aiko N. Matsumoto

**Affiliations:** 1 » Degree in Dentistry - University of Passo Fundo (UPF), Passo Fundo, Brazil. » Specialization in Radiology and Imaging - Federal University of Rio de Janeiro (UFRJ), Rio de Janeiro, Brazil. » Master’s Degree and Doctorate in Dentistry (Orthodontics) - UFRJ, Rio de Janeiro, Brazil. » Post Doctorate, Case Western Reserve University (CWRU), Cleveland, OH. » Associate Professor, Orthodontics - UFRJ, Rio de Janeiro, Brazil. » Director, Brazilian Board of Orthodontics and Facial Orthopedics (BBO). » Reviewer of: European Journal of Orthodontics (EJO), Clinical Oral Implants Research (COIR), International Journal of Oral Science (IJOS), Brazilian Oral Research (BOR), Revista Clínica de Ortodontia Dental Press, and Progress in Orthodontics. » Scientific Consultant, INP Biomedical (implant systems) and Click Aligner.; 2 » Master’s Degree in Orthodontics, Federal University of Rio de Janeiro (FO-UFRJ), Rio de Janeiro, Brazil. » Doctorate in Orthodontics, UFRJ/UCLA. » Reviewer, AJODO. » Professor, Graduate Program, Federal University of Paraná (UFPR), Curitiba, Brazil.; 3 » Associate Professor of Orthodontics, School of Dentistry, Federal University of Goiás (FO-UFG), Brazil. » Professor, Graduate Program of Dentistry (Master and Doctorate), FO-UFG. » Doctorate in Orthodontics, School of Dentistry, Universidade de São Paulo (FOUSP), São Paulo, Brazil. » Member of the Board of Directors of the Brazilian Board of Orthodontics and Facial Orthopedics (BBO).; 4 » Master’s Degree and Doctorate in Orthodontics, FO-UFRJ, Rio de Janeiro, Brazil. » Full Professor of Orthodontics, School of Dentistry, Pontifícia Universidade Católica do Rio Grande do Sul (PUCRS), Porto Alegre, Brazil. » Coordinator of the Graduate Specialization Course, Brazilian Dental Association - Rio Grande do Sul Chapter (ABO/RS). Porto Alegre, Brazil.; 5 » Master’s Degree in Orthodontics, UFRJ, Rio de Janeiro, Brazil. » Diplomate, Brazilian Board of Orthodontics and Facial Orthopedics (BBO).; 6 » Master’s Degree and Doctorate in Orthodontics, FO-UFRJ, Rio de Janeiro, Brazil. » Associate Professor Class 2 of Orthodontics, School of Dentistry of Ribeirão Preto, USP (FORP-USP), Ribeirão Preto, Brazil. » Coordinator, Graduate Program in Orthodontics, FORP-USP. » Professor, Graduate Program (Master and Doctorate), FORP-USP. » Member of the Board of Directors of the Brazilian Board of Orthodontics and Facial Orthopedics (BBO).

**Figure:**
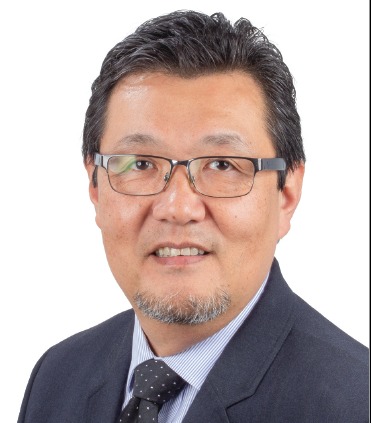

Those who do not know Lincoln Issamu Nojima may have the impression that he is a sedate professor of Orthodontics. However, this great professional has struggled and endured a difficult journey to become the orthodontist that he is today. In the beginning of his career, as he had no dependable advisor, he attended several short-duration courses. He took his first course on typodont in 1989. However, unsatisfied with his learning up to that time, he sought to improve his education by taking other courses to achieve the goal of being a good orthodontist. He even took a course at the Tweed Foundation in 1994. Until one day, his friend Geraldo Queiroz suggested that he should take the Orthodontics course at the Federal University of Rio de Janeiro (UFRJ), which he did. He went on to take a Master’s Degree (1995-1996) and a Doctorate (1999-2004) and started working as a professor in the Department of Orthodontics of UFRJ in 1997. In his search for more knowledge, he had the opportunity to take a Post Doctorate (2011-2012) in the Case Western Reserve University, Cleveland, OH, where he is currently a Visiting Associate Professor at the same Department of Orthodontics. He works in a research project in partnership with the Department of Orthodontics of the Yonsei University, Seoul, South Korea, with which he has a cooperation agreement. His line of clinical research investigates current topics, such as miniscrew-assisted rapid palatal expansion (MARPE), mini-implants, cone-beam computed tomography, orthodontic aligners and indirect orthodontic bracket bonding. However, during this promising, successful journey, he was not alone: he has had the unconditional support of his wife, Matilde, with whom he raised a beautiful family, with his sons Vitor Kenji and Pedro Eiji. A devoted father and husband, he loves to practice Kendo after a busy working day. He also enjoys fishing and walking with his family on weekends. It is an honor to have been invited to coordinate his interview, particularly because of the chance to talk about a serious, devoted, intelligent professional that is extremely proactive and an example to be followed. 

Quem não conhece Lincoln Issamu Nojima pode ter a impressão de que se trata de um pacato professor de Ortodontia. Entretanto, esse grande profissional lutou e percorreu uma difícil trajetória para conseguir ser o ortodontista que é hoje. No início de sua carreira, sem uma orientação segura e responsável, frequentou vários cursos de curta duração. Fez seu primeiro curso de typodont em 1989; entretanto, não satisfeito com o conhecimento adquirido, buscou melhorar sua formação em outros cursos para alcançar o objetivo de ser um bom ortodontista. Chegou, inclusive, a fazer curso na Tweed Foundation em 1994. Até que seu amigo Geraldo Queiroz sugeriu que ele deveria fazer o Curso de Ortodontia na UFRJ, e assim o fez. Cursou o Mestrado (1995-1996) e o Doutorado (1999-2004), e ingressou como docente no Departamento de Ortodontia da UFRJ em 1997. Na busca por mais conhecimento, teve a oportunidade de fazer o pós-doutorado (2011-2012) na Case Western Reserve University (Cleveland/OH, EUA), onde atualmente é Visiting Associate Professor no mesmo Departamento de Ortodontia. Desenvolve projetos de pesquisa em parceria com o Departamento de Ortodontia da *Yonsei University* (Seul, Coreia do Sul), com o qual mantém acordo de cooperação. Sua linha de pesquisa clínica está fundamentada em temas atuais, como MARPE, mini-implantes, tomografia computadorizada de feixe cônico, alinhadores ortodônticos e colagem indireta de braquetes ortodônticos. Entretanto, durante toda essa trajetória promissora e de sucesso, não esteve sozinho: tem recebido o suporte e o apoio incondicional de Matilde, sua companheira, com quem formou uma linda família, com os filhos Vitor Kenji e Pedro Eiji. Pai e esposo dedicado, gosta muito de praticar Kendo após um dia atribulado de trabalho. Curte, também, pescar e fazer caminhada nos fins de semana, com sua família. Ser convidada para coordenar sua entrevista muito me honra, sobretudo por permitir falar de um profissional sério, dedicado, inteligente, extremamente proativo, um exemplo a ser seguido! 

Mirian Aiko Nakane Matsumoto (Interview coordinator / coordenadora da entrevista)

Comparing conventional and digital indirect bonding, what are the advantages and disadvantages of each of these techniques? (Marco Schroeder)

Indirect bonding is a well-disseminated technique in countries such as the United States, Japan, South Korea, England and Australia, because it reduces chair time and, consequently, improves patient comfort. Several studies have confirmed indirect bonding accuracy in positioning brackets, reducing the need for rebonding and second order bends.

I have been using indirect bonding for some years; however, I improved its use when I met Dr. Ronald Heiber during my postdoctoral fellowship in the Case Western Reserve University (CWRU). Dr. Heiber used a rather interesting technique of bracket positioning guided by reference lines drawn from the marginal ridges of the posterior teeth. This procedure is quite accurate (Fig. 1), as it determines bracket positioning using the references of the occlusal contacts, and not the cusp tips, which have individual anatomic variations. Therefore, it is not necessary to use an instrument to position the brackets in the posterior area, because the parameter used is the morphology of each individual tooth.^1^

**Figure 1:**
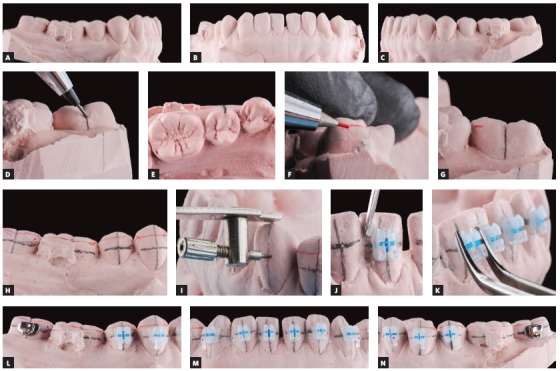
Sequence of planning for conventional indirect bonding: A, B, C) initial dental casts in type IV dental stone; D, E) vertical tracings of individual tooth axes. F, G) red horizontal lines drawn to determine cusp height according to marginal ridges; H, I) additional black horizontal line proportional to the distance of marginal ridges, used so that brackets can be positioned vertically without interfering with occlusion; J, K, L, M, N) after isolation of working models, brackets are bonded using conventional resin, to customize bracket base (Source: Nojima, Araújo, Alves Júnior,^1^ 2015).

In clinical routine, I have used both types of indirect bonding techniques, which we may call conventional, using working models, and digital, or virtual. Both are efficient for their purposes. If we analyze planning, we see that, in the conventional technique, the well-trained clinical dentist does not need more than 10 minutes to draw the reference tracings that will be used for the positioning of brackets. In contrast, the digital technique demands a longer time because of the virtual setup, that is, the dentist should place teeth correctly on the final configuration of the orthodontic treatment to position brackets, which adds accuracy to the procedure. During tray manufacture, the orthodontist, laboratory technician or assistant that uses the conventional technique needs a longer time to prepare the transfer guides. In this aspect, the digital technique has an advantage, because the guides are manufactured using a 3D printer, which speeds up the process in the office. 

However, I believe that the cost and benefit balance of the conventional technique is more advantageous than that of the digital technique, which makes it a more viable alternative. Indirect digital bonding is interesting for clinics with a larger number of patients. Individual dentists would have to have the financial resources to acquire the equipment, which includes intraoral or desktop scanner, 3D printer and specific software, and they would also have to hire specialized personnel. Another way to make its use possible would be outsourcing it to companies working with the digital technique, but, depending on the demand in each office, this may also have a high cost. Summing up: both indirect bonding techniques are functional and accurate; however, choices will depend on the demand in each office or orthodontic clinic.

In your large experience, what is the difference regarding bonding precision and shear bond strength of the digital and conventional indirect bonding techniques? Based on your studies in this area, which bonding material would you recommend for the indirect technique today? (Daniel Brunetto)

After the CAD-CAM systems were introduced in Orthodontics, customized brackets and virtual setups ensure greater precision for orthodontic appliance installation, mainly because of the visualization of results and the almost ideal positioning of brackets (Fig 2). However, the cost and benefit ratio of this system is still greater than that of the conventional systems that use casts, as discussed above. But I believe in the global trend of making free software available, with payments only when a case is exported.

**Figure 2:**
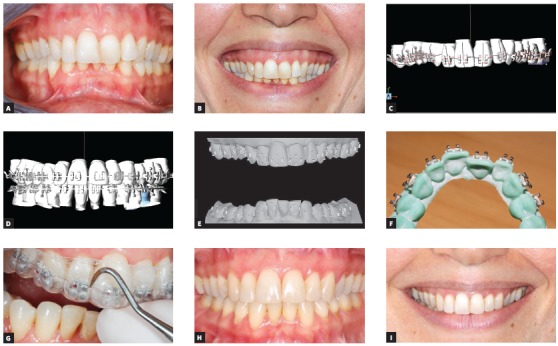
45-year-old female patient with Class II malocclusion and 5-mm overjet; main complaint was anterior crowding; previous orthodontic treatment included extraction of mandibular right central incisor. Patient underwent ortho-surgical treatment, with virtual indirect bonding and extraction of other mandibular central incisor. A, B) Baseline frontal views; C, D, E) digital setup with planning of fixed orthodontic appliance installation and production of bonding guides in STL format; F) bracket bonding over guide lines in prototyped models; G) manufacture of transfer guides in double tray and indirect bonding; H, I) finishing of clinical case.

Both indirect bonding techniques have shear bond strength that is equal to or greater than that of direct bonding.^2^ ^,^ ^3^ Digital indirect bonding uses prototyped guides, a technique in which the brackets are positioned directly according to the tie-wings and transferred to the oral cavity. Conventional light-curing resins for direct bracket bonding are used for that. Excess resin should be removed after the guide has been dislodged. In the conventional technique, bonding with light-curing resins is performed directly on the working models that have been previously isolated.^1^ This way, the tooth surface is customized, and shear bond strength is increased. Queiróz Tavares et al^2^ found that flowable resins are an excellent bonding material for these cases, and its bond strength is equal to or greater than that obtained with conventional direct bonding. Chemically cured resins type A or B used for indirect bonding are no longer indicated for this purpose because of their low shear bond strength.^2^

You have a strong educational background in Orthodontics, enriched by a time abroad in CWRU and complementary training in Radiology. How and in what aspects have these experiences and background affected your clinical practice? (Luciane Menezes)

The School of Orthodontics founded by Professor José Édimo Soares Martins trained professionals that, in turn, created other excellent Postgraduate Programs in Brazil. I really believe that my education in Orthodontics at the Federal University of Rio de Janeiro (UFRJ) has given me a rather solid knowledge basis. Basic principles are essential for orthodontists to navigate turbulent seas, particularly when there is a diversity of techniques and treatment systems developed by orthodontic companies. 

The Bolton-Brush Growth Study Center at CWRU has had an important role in global Orthodontics, as it established the concepts of craniofacial growth. Some names of excellence in Orthodontics are associated with this renowned study center, such as Holly Broadbent, Donald Enlow and Idell Pyle. As the CWRU Department of Orthodontics was one of the first to adopt the use of cone-beam computed tomography (CBCT), I had the opportunity to work side by side with excellent professors, such as Juan Martin Palomo, Mark Hans and Lysle Johnston. The 18 months I spent at CWRU opened my horizons and my perspectives to Orthodontics in a less romantic and more realistic way. Part of my traditional ties was broken, experiencing the work of other professionals. I have always been fond of new technologies; after all, I was a pioneer in the use of digital photography in Orthodontics and skeletal anchorage in Brazil. When I realized that I was in the Department of Orthodontics associated with the Bolton-Brush Growth Study Center - where the study of craniofacial growth was born, and which already adopted CBCT and digital models as diagnostic tools for some conditions as early as in 2011-2012 -, I was definitely fascinated with the opportunity to share that experience and bring those innovations to orthodontic practice. American Orthodontics is very rich, and new technologies entered my clinical and academic life without curtailing the quality of what I do. 

In daily clinical practice, which are the main applications of CBCT? What is its role in planning the use of miniscrew-assisted rapid palatal expansion (MARPE)? What CBCT protocol do you use in these cases? (Daniel Brunetto)

The use of CBCT makes diagnoses much more accurate and reliable. The 3D reconstructions provide an overall panoramic view and help locate areas of interest, as well as allow to place the patient’s head in different positions, for the analysis of CT slices. The knowledge of the three basic CBCT planes - axial, coronal and sagittal - is fundamental for the examination of anatomical sections. One of the ways to work with CBCT is to request prints of the slices of regions of interest, so that the radiologist may interpret and report findings. In addition, it is possible to perform evaluations directly on tomographic slices. Currently, there is a plethora of free software, called viewers, that may facilitate clinical routine in Orthodontics when DICOM files are requested for analysis. 

The indications of CBCT are varied, but I see that, in Orthodontics, this imaging study is fundamental in some cases. Patients with facial asymmetry may present with a skeletal compromise associated with changes in the temporomandibular joint. I usually indicate CBCT scanning of the whole face - in these cases, I do not request complementary radiographs for the diagnosis, because I am able to generate reconstructions of panoramic and lateral cephalometric radiographs using the DICOM file. At the same level of importance, I recommend CBCT scanning of the whole face for patients with an indication of orthognathic surgery. For individuals with more localized changes, such as impacted teeth, bone fenestrations and bone defects, I may indicate CBCT scanning of the maxilla or mandible (Fig 3). 

**Figure 3:**
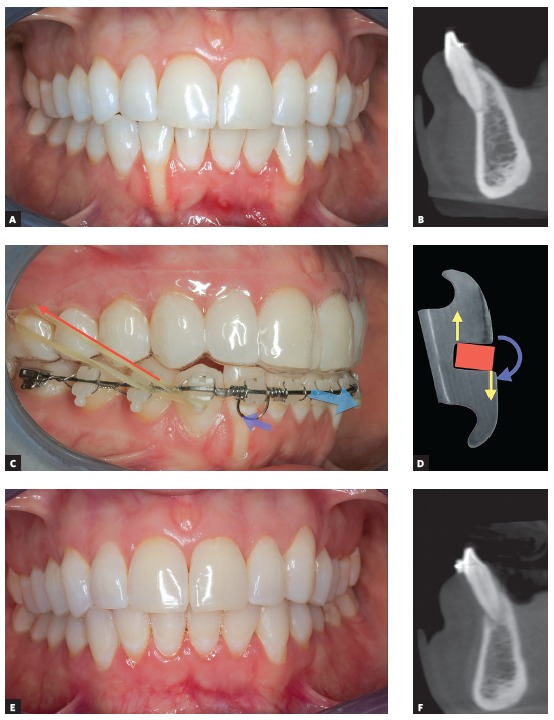
A) 25-year-old female patient referred by periodontist after identification of gingival retraction in tooth #43; B) CBCT was required to visualize the degree of bone fenestration; C, D) orthodontic mechanics using 0.014-in stainless steel torqueing spring in passive TMA archwire, with active lingual root torque on tooth #42. To avoid protrusion of mandibular incisors, Class II elastics were anchored to passive 0.8-mm maxillary EVA aligner; E, F) active orthodontic treatment after 90 days; CT scan shows tooth root moved into mandibular symphysis. Periodontal plastic surgery with connective tissue graft (Dra Daniela Jusan) to recover area; 9 months after orthodontic treatment.

In cases of MARPE, CBCT of the maxilla is essential for the success of the technique, as the scans are used to define the position of the jackscrew and correctly select the length of miniscrews for bicortical anchorage. The protocol of CBCT acquisition in planning of MARPE cases should ensure that the field of view (FOV) is small, that is, limited to the maxilla, so that a more detailed view of the bone structures is acquired. As measurements are also made on the palatal mucosa, it is fundamental to instruct the patient to move away the tongue from the palate and to open the mouth during CBCT scanning. This will ensure a better visualization of soft tissue thickness in the region of the palate.

What is your opinion about the use of new diagnostic imaging tools, such as CBCT and digital models, in orthodontic practice? (Luciane Menezes)

These digital diagnostic tools have already been incorporated into the daily routine of orthodontists today. I have seen this trend for over 10 years. It is impossible not to use digital tools in orthodontic diagnosis and planning in several clinical cases, as discussed above. Digital models have already replaced conventional working models in numerous clinical cases. A typical example is the digital setup, incorporated as a diagnostic resource in planning of cases that will use fixed orthodontic appliances, as well as those with an indication of orthodontic aligners. The use of digital systems is much faster and cleaner, even if they have some minor flaws. Other typical cases are those of multidisciplinary planning, such as in Orthognathic Surgery, which depends on CBCT scans and digital models for the manufacture of surgical guides. At the same time, Orthodontics, Implantology and Oral Rehabilitation require the use of these digital resources to define and evaluate tooth movement, osseointegrated implant positioning and impressions for provisionals. 

Digital models are crucial in planning of orthodontic aligners, bracket manufacture and use of customized orthodontic archwires in the lingual and buccal systems. Therefore, indirect bonding systems also benefit from the use of this technology. Another point is the work with laboratories, where communications are gradually becoming fully digital. Applications and software are increasingly more user-friendly and low cost. In contrast, hardware, such as 3D printers and scanners, remain very expensive for most Brazilian orthodontists. But I assure you that, as part of the constant evolution that we have experienced, there is no escaping these technologies, as orthodontists will, otherwise, be doomed to oblivion.

In your clinical experience and based on all your learnings at Yonsei University, South Korea, what are the main factors that may affect the success of MARPE in skeletally mature patients? (Daniel Brunetto)

This is a recurrent question among orthodontists. The Yonsei University Group, led by Professor Kee Joon-Lee, has had a success rate of 86.96% for MARPE in young adults, which, in a sample of 69 patients, meant a rate of 60 midpalatal suture openings.^4^ This seems quite promising, but there is still a percentage of cases for which this technique is not successful. We should not forget that the greatest resistance to midpalatal suture opening is found in the posterior region of the palatine bone, in the pterygopalatine suture (Fig 4A). The transverse and anteroposterior location of the jackscrew in the palate is fundamental for the success of this technique.^5^ Therefore, the use of CT scans for the correct selection of miniscrews should be part of the orthodontist’s armamentarium when planning this type of case.^6^ Bicortical anchorage is fundamental for the stability of miniscrews and their resistance to forces of midpalatal suture opening and displacement of the pterygopalatine suture (Fig 4B). 

**Figure 4:**
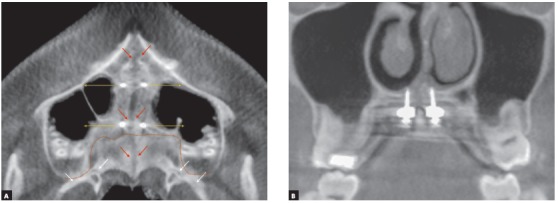
A) CBCT axial slice 15 days after MARPE was completed. Direction of the forces applied to miniscrews (yellow arrows), hypodense area showing opening of midpalatal suture (red arrows) and pterygopalatine suture (white arrows). B) Coronal CBCT slice of posterior MARPE region, showing bicortical anchorage.

Another important factor that may affect the success rate of MARPE is the degree of maxillary deficiency. Individuals with a hyperdivergent facial pattern, with marked transverse deficiency and high-arched palate, may experience complications of this technique. In these conditions, the palate mucosa has a thick, poorly keratinized layer in the paramedian area, which increases the distance between the bone and the miniscrew support on the jackscrew, thus reducing miniscrew stability.

What is the maximum chronological age, or maturation stage, for an indication of MARPE? In other words, what is the limit between the indication of MARPE and surgically-assisted rapid palatal expansion (SARPE)? (José Valladares, Marco Schroeder)

I prefer not to talk about a maximum age for the indication of the use of MARPE, because its success is more closely associated with the shape of the palate and the level of maturation of the midpalatal suture. Some studies tried to identify the stage of skeletal maturation using radiographs and CBCT scans; however, some sutures are particularly difficult to classify. It is important to make it clear to the skeletally mature patient with maxillary transverse deficiency that there is a percentage of failure when MARPE is used.^4^ In these cases, SARPE would have to be used eventually. In general, I still prefer to first indicate MARPE for individuals with moderate transverse deficiency instead of indicating SARPE from the beginning. 

Individuals older than 40 or 50 years have a greater resistance to midpalatal suture opening, but MARPE may have a favorable response in these cases. In a research for a Doctorate thesis in Yonsei University, which originated studies that are about to be published, Cunha et al.^7^ found interesting facts about the midpalatal and pterygopalatine sutures. Necropsy specimens collected from eight cadavers of individuals aged 72 to 88 years were analyzed using micro-CT, and sutures were not yet fully obliterated, despite the great complexity of the bone interdigitations in the midpalatal, transverse and pterygopalatine sutures.^7^

Currently, jackscrews are adjustable to palate depth. However, individuals with severe transverse deficiency and a high-arched palate have a thickening of the palate mucosa and, consequently, a greater distance between the site of force application to the miniscrew and the bone anchorage. In cases of poor bone quality, this may generate great miniscrew instability. 

You are one of the pioneers in the use of MARPE in Brazil. What are the differences between MARPE, conventional rapid palatal expansion (RPE) and SARPE? (Luciane Menezes)

All the three techniques are important and should be indicated according to strict clinical criteria. However, the way dental and skeletal structures behave in maxillary expansion gives rise to discussions. In the case of RPE, indicated for very young individuals, midpalatal suture opening occurs at about 30% of jackscrew activation.^8^ This is explained by the fact that the line of force application is located in the anchorage teeth, below the point of greater resistance, located at the midpalatal and pterygomaxillary sutures. Because of that, there is a buccal inclination of the alveolar processes, something quite common in children with mixed dentition and at the early phase of permanent dentition (Fig 5A). 

**Figure 5:**
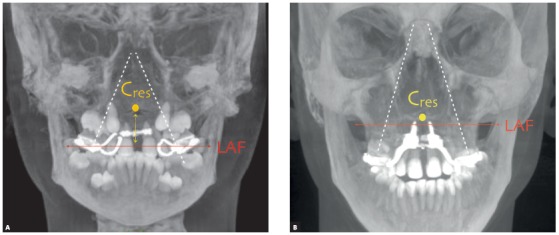
Thick CBCT coronal slice (160 mm). A) Patient after RPE; red arrow indicates line of action of force (LAF) at the level of anchorage teeth, at a certain distance from the center of resistance (Cres), in yellow. White dashed line indicates opening of maxillary bones. B) Patient undergoing MARPE: red arrow indicates line of action of force; center of resistance in yellow. Force is applied at a point very close to center of resistance of midpalatal suture. Angle of opening of maxillary bones (white dashed line) is less accentuated.

In the case of MARPE, forces are applied directly to the miniscrews placed in the paramedian area. Therefore, the line of force application runs through the area of greater resistance and promotes uniform opening and less inclination of dentoalveolar structures and, consequently, of maxillary bones (Fig 5B). This movement expands the nasomaxillary area and substantially improves airflow, particularly in those individuals with obstructive sleep apnea.^9^

SARPE is a surgical expansion with Hyrax-type appliances that are exclusively tooth-borne. It is indicated for patients with severe transverse deficiency, with good predictability of results. However, differently from MARPE, SARPE does not increase airflow after expansion,^10^ because the osteotomy is performed below the nasal cavity. 

In MARPE, what is the strategy to place miniscrews using bicortical anchorage, which uses the palatal cortical bone and the floor of the nasal cavity, preserving the nasal mucosa? What clinical management should be adopted in case of nasal mucosa perforation? (José Valladares Neto)

One of the requisites for the success of MARPE is bicortical anchorage. However, care should be taken not to perforate the total thickness of the nasal mucosa to avoid possible complications, such as bleeding and even rhinosinusitis. Therefore, dentists should plan the placement of the jackscrew and the selection of miniscrew length accurately. The use of CBCT scans to measure bone and palate mucosa height is fundamental for the success of this technique and for the prevention of possible complications. Measurements are usually made on the coronal view of the region in which the miniscrew fixation rings connects to the jackscrew. This procedure may be facilitated if you have the appliance and the working model in your hands. Another important measurements are the height of miniscrew fixation rings and the distance from the palatal surface, measured directly on the working models using a millimeter probe. We wrote a detailed article about the protocol for selection of miniscrews used with MARPE.^6^

What is your experience with maxillary expansion using exclusive bone-borne anchorage? (José Valladares Neto)

My experience with this technique alone is very little, because I believe that the technique that includes tooth-borne systems is more functional. You see, tooth-borne MARPE grants greater stability to the system and provides adequate spacing. When manufacturing the expander in the laboratory, a distance of 1 mm to 2 mm between miniscrew fixation ring and palatal surface, as well as some space from the lateral arms fixed to the bands, should be kept. This is an important step to avoid that the appliance come closer to the soft tissue, which may initiate a local inflammatory response and, because of that, lead to the need to remove the miniscrews and even all the system in some cases. By analogy, we may say that teeth work as two bridge pillars that ensure adequate separation, which is very difficult to achieve in the case of exclusively bone-borne expanders. Another important point about bone-borne anchorage is the clinical difficulty to insert miniscrews in this type of system, which requires the manufacture of guides that help stabilize the expander. However, the use of an expander with skeletal anchorage only should not be ruled out in selected cases. After expansion using MARPE, during retention, the arms that connect the jackscrew to the tooth may be cut, and the bands, removed. This adds versatility to the system, so that teeth may be moved as needed while skeletal expansion is stabilized by miniscrews.

You are an outstanding researcher of the mechanical and physical properties of miniscrews and has even designed devices for large companies. However, today, what factor do you see as the most important for success rates, and what are the main factors responsible for failure of miniscrews placed in alveolar bone? (Daniel Brunetto)

I would classify the success and failure of all types of miniscrews into some categories, but the learning curve helps increase the rate of success for the use of skeletal anchorage. In the cases of surgical placement, miniscrew design is important. That is, self-drilling miniscrews in which the tip thread has a greater angle have a better cutting ability and, therefore, provide better primary stability. A dentist’s experience is essential to define how much force to apply when placing miniscrews and to assess the quality of the bone to determine when appliance placement should be stabilized and, after that, to calculate immediate loads. Bone quality also affects miniscrew stability. It is evident that the quality of cortical bone is very poor in edentulous alveolar areas, which contributes to the loss of appliance stability. Areas close to frenula and muscle insertions are also factors that may lead to miniscrew failure.

Biomechanic knowledge of fundamental concepts, such as line of action of a force, resolution of vectors and the relationship between momentum and force, should be part of the wealth of information and knowledge held by an orthodontist that uses skeletal anchorage in daily clinical routine. 

In cases of posterior teeth retraction (over 3 mm) using skeletal anchorage, regardless of type - intra-alveolar miniscrews, extra-alveolar miniscrews, or miniplates -, do you prefer to use partial or en-masse retraction? (Daniel Brunetto)

Dental arch retraction with no-extraction is currently in vogue. I am not a fan of “fads”, and I deeply believe in scientific principles and clinical experience. I believe that each case is unique and depends on diagnosis and planning to achieve the adequate parameters to correct malocclusion. Moreover, I am used to sharing the advantages and disadvantages of possible treatment approaches with the patients and their guardians, so that we share responsibility. Now, about orthodontic mechanics, I have already used the three skeletal anchorage systems in partial and in en-masse retractions. However, the main criterion for retraction cases is, once again, planning, including important aspects to be analyzed. In cases of anterior deficiency, due to protrusion or severe crowding, I prefer a faster treatment, with the extraction of premolars and creation of spaces close to the region where the main problem is - in this case, the anterior region. I use both sliding mechanics with stainless steel rectangular archwires, which have good formability and provide more uniform and predictable tooth movements; and loop archwires. In both situations, I use interradicular miniscrews for the retraction of the six anterior teeth. For the cases in which the deficiency is less severe, I use dental arch retraction with interradicular miniscrews, extra-alveolar miniscrews or miniplates. In these clinical cases, I perform the movement in two steps, that is: I begin with the retraction of the posterior region and, after that, of the anterior region. This way, tooth movement is more gradual, uniform and predictable. I am not in favor of great retractions, because they require much more care due to the anatomy of the region. The maxillary arch is easier to diagnose, as the orthodontist may measure the amount of space in the region of the tuberosity. In the mandibular arch, the anterior border of the mandibular ramus limits the space available. For many years, we believed that the ramus was the anatomic obstruction that blocked retraction in the posterior region of the mandible. After the advent of CBCT, we found that the lingual cortical bone of the mandibular body is the limiting factor for this movement, as the apical third of the root of the permanent mandibular second molar has its anchorage in this bone, and blocks its retraction.^11^ Accentuated lingual tipping movements and distal tipping of the crown are clinical signs that the root has touched the lingual cortical bone of the mandibular body. 

Skeletal anchorage may be seen as an evolution of Orthodontics that originated in the need to follow the changes in current society. What is your opinion about the other skeletal anchorage devices available in the orthodontic market, that is, extra-alveolar miniscrews and miniplates, in comparison with intra-alveolar miniscrews? (Mirian Matsumoto)

I have been working with miniscrews for about 20 years. I have followed all the evolution of skeletal anchorage. With the advent of extra-alveolar miniscrews, interradicular miniscrews begin to be called intra-alveolar by some professionals. But I still prefer the term interradicular miniscrews. Each of the three temporary anchorage devices mentioned in your question have positive and negative points, as well as clinical indications and contraindications. I have always used interradicular miniscrews since I started working with skeletal anchorage, and, based on growing support from literature, I have also worked with extra-alveolar miniscrews. 

The interradicular devices are excellent for minor sagittal movements, or for greater sagittal movements performed in two stages. I also indicate its clinical use for transverse inclination, such as in cases of posterior crossbite, and indirect anchorage. For single or en-masse posterior tooth intrusion and contraction of the dental arch, I prefer to place miniscrews in the palate, including the paramedian or midpalatal suture region, both classified as extra-alveolar areas. After the introduction of the miniscrews for the infrazygomatic crest and the posterior area of the mandibular body, buccal to the roots of the mandibular molars, that is, in the buccal shelf - also classified as an extra-alveolar area -, the use of these anchorage devices for large sagittal movements and maxillary intrusion became more widely adopted. I believe that the placement of miniscrews in the mandibular arch does not lead to any problems because of the anatomic bone volume in the external oblique ridge of the mandible. Vargas et al.^12^ found that the greatest bone thickness is at the level of the distal root of the mandibular second molar, and bone thickness in the buccal shelf is greater in individuals with a very small mandibular angle.^11^ However, Baumgaertel and Hans^13^ found that the bone in infrazygomatic crest is not very thick. I agree, therefore, that the use of maxillary extra-alveolar miniscrews should be a little more carefully decided. Special care should be taken because of the maxillary sinus. The perforation of the internal mucosa of the maxillary sinus associated with a possible inflammation in response to miniscrews, which is not rare, may lead to the development of sinusitis. No report of this complication has been made, but I should stress that I also do not see many control imaging studies being performed after the placement of miniscrews in this anatomic area by clinical dentists that use this technique routinely. The results of studies that we have conducted at UFRJ to evaluate bone thickness in the area of the infrazygomatic crest corroborate the evidence that it has a thin cortical bone.^12^ ^,^ ^14^ Baumgaertel and Hans^13^ concluded that miniscrews 6.00 mm or longer usually result in perforation of the maxillary sinus. Therefore, you cannot be too careful here. 

Miniplates are excellent temporary anchorage devices. In use for many years, they provide good resistance to the application of orthodontic forces. When they emerge into the oral cavity, through the alveolar mucosa, they may cause some discomfort. However, more anatomic miniplates have been introduced in the market, and they emerge in the area of attached gingiva, with good clinical results in young individuals with Class III malocclusion. I have used miniplates for the interception of these malocclusions in individuals in the late mixed or early permanent dentition phase following the protocol described by De Clerck^15^, with quite promising results. Miniplates are also indicated for retraction of the maxillary and mandibular arches as part of coherent orthodontic planning, respecting the limits of biological principles.

## References

[1] Nojima LI, Araújo AS, Alves M (2015). Indirect orthodontic bonding: a modified technique for improved efficiency and precision. Dental Press J Orthod.

[2] Tavares MLQ, Elias CN, Nojima LI (2018). Effects of different primers on indirect orthodontic bonding: Shear bond strength, color change, and enamel roughness. Korean J Orthod.

[3] Sha HN, Choi SH, Yu HS, Hwang CJ, Cha JY, Kim KM (2018). Debonding force and shear bond strength of an array of CAD/CAM-based customized orthodontic brackets, placed by indirect bonding - An in vitro study. PLoS One.

[4] Choi SH, Shi KK, Cha JY, Park YC, Lee KJ (2016). 2016 Nonsurgical miniscrew-assisted rapid maxillary expansion results in acceptable stability in young adults. Angle Orthod.

[5] Brunetto DP, Sant'Anna EF, Machado AW, Moon W (2017). Non-surgical treatment of transverse deficiency in adults using Microimplant-assisted Rapid Palatal Expansion (MARPE). Dental Press J Orthod.

[6] Nojima LI, Nojima MDCG, Cunha ACD, Guss NO, Sant'Anna EF (2018). Mini-implant selection protocol applied to MARPE. Dental Press J Orthod.

[7] Cunha AC, Nojima LI, Nojima MCG (2017). Expansão maxilar assistida por mini-implantes: estudo do complexo sutural craniofacial sob perspectivas clínicas e microestruturais.

[8] Baratieri C, Nojima LI, Alves M, Souza MMG, Nojima MG (2010). Transverse effects of rapid maxillary expansion in Class II malocclusion patients: a Cone-Beam Computed Tomography study. Dental Press J Orthod.

[9] Hur JS, Kim HH, Choi JY, Suh SH, Baek SH (2017). Investigation of the effects of miniscrew-assisted rapid palatal expansion on airflow in the upper airway of an adult patient with obstructive sleep apnea syndrome using computational fluid-structure interaction analysis. Korean J Orthod.

[10] Camps-Perepérez I, Guijarro-Martínez R, Peiró-Guijarro MA, Hernández-Alfaro F (2017). The value of cone beam computed tomography imaging in surgically assisted rapid palatal expansion: a systematic review of the literature. Int J Oral Maxillofac Surg.

[11] Kim SJ, Choi TH, Baik HS, Park YC, Lee KJ (2014). Mandibular posterior anatomic limit for molar distalization. Am J Orthod Dentofacial Orthop.

[12] Vargas EOA, Nojima LI, Lima RL (2019). Avaliação da espessura óssea do shelf mandibular e da crista infrazigomática em indivíduos com diferentes alturas faciais verticais.

[13] Baumgaertel S, Hans MG (2009). Assessment of infrazygomatic bone depth for mini-screw insertion. Clin Oral Implants Res.

[14] Bernardino I, Ferreira JB, Nojima LI (2017). Avaliação da espessura óssea da crista infrazigomática para a inserção de mini-implantes.

[15] De Clerck HJ, Cornelis MA, Cevidanes LH, Heymann GC, Tulloch CJ (2009). Orthopedic traction of the maxilla with miniplates a new perspective for treatment of midface deficiency. J Oral Maxillofac Surg.

